# Medical and Physical Intelligence

**Published:** 1826-06

**Authors:** 


					515
MEDICAL AND PHYSICAL INTELLIGENCE.'
COMPARATIVE ANATOMY.
1. Dissection of an Ourang Outang.?The skin was attached very
closely to the body at all parts, particularly on the face, hands, elbows,
and soles of the feet. He had no cutaneous muscles except the platisnia
myoides: this was not connected on its inner surface, but formed a
large pouch, extending from the chin to the sternum, continuing round
to the sides of the neck. It was supposed by those who saw him to be
a receptacle for food. This was not, however, its purpose ; for it com-
municated with the larynx, and not with the pharynx, as will be de-
scribed when speaking of those parts.
The abdomen presented a view so similar to the human, that it
required some attention to note any peculiarities. The omentum was
small; lying high up the intestines, coloured with bile, as were the
bowels generally. The peritoneal folds were very strong, particularly
the ligaments of the liver, the mesentery, &c.; the caput coli was also
strongly confined to its place. The spermatic cord received its parts,
and passed obliquely under the muscles, and came out at Pouparl's li-
gament, as in man. The proportion of the small to the large intestines
was about the same as in the human subject. The arch and sigmoid
flexure of the colon exceedingly resembled the human. He had the
appendix vermiformis very long, measuring four inches: this 1 found
full of small stones and some pieces of egg-shell, together with liquid
faeces. The large intestines were found loaded with indurated faeces,
from the caput coli to the extremity of the rectum. The stomach, in
situation and figure, was like a man's; its cardiac orifice was perhaps
smaller, and the pylorus larger. Its dimensions were, when inflated,
from one orifice to the other, round the fundus, ten and. a half
inches; across, it measured three inches: it was nine inches in
circumference round the fundus. The spleen was attached by the
vasa brevia, and, in colour, size, and situation, accorded to man's.
The liver was very much like ours, of a deep red colour, and
divided into two lobes; but the fissure was not quite so distinct: in
connexion with the other viscera, it appeared exceedingly like the hu-
man. The gall-bladder was much longer, and smaller round, and was
found full of dark inspissated bile, which could with difficulty be
crowded along the duct. The pancreas laid upon the spine, as in man.
These had all their orifices opening into the bowels, in the same way as
the human. The kidneys did not present any difference, excepting that
the renal capsules were larger. The bladder was small, containing,
when full, about two gills. The urethra, prostate gland, vesiculae,&c.
were situated like the human. The prepuce, glans, &c. were like the
human, but small. The organs of the chest resembled the human in
size, figure, and situation. The lungs did not present quite so much
difference on the two sides as in man; that of the left being nearly of
the same size with the right, carrying the heart more towards the centre
of the thorax. The lungs were not so distinctly divided into lobes;
516 Medical and Physical Intelligence.
they were very sound and healthy in appearance. The heart was eonr-
cal, like man's, and in every respect resembled the human. The areb
of the aorta, and the descending aorta, were small in proportion to the
size of the heart. The right subclavian, right and left carotid arteries,
ail arose from the arteria innominata ; the left subclavian rose sepa-
rately, near the base of this. The pericardium was connected exten-
sively to the diaphragm, which was very large and strong. The chest
was divided by the mediastinum, and the thymus gland laid between its
sides.
The mouth and fauces resembled the human, except in dimensions,
being much longer from front to rear. The velum palati was without
the uvula, but broader and more lax. The body which answered the
purpose of the uvula was situated on the posterior surface of the ve-
lum; and, when this was forced backwards, exactly closed the posterior
entrance of the nose. The glottis and epiglottis resembled the human.
The os hyoides and cartilages of the larynx were much as in man. Be-
tween the os hyoides and the thyroid cartilage, there were on each side
two openings, about a quarter of an inch in diameter, leading into the
larynx, and coming out at the base of this cartilage. A valve played at
the inferior opening, preventing the passage of an instrument downward;
but it passed easily upwards into the pouch on the neck, which has been
mentioned. This pouch the animal could inflate at pleasure; for what
purpose I do not know. One use might be, when inflated, to assist in
supporting him when swimming.
The brain weighed nine ounces and three-quarters. The nerves
arose from this in the same manner as the human, and took their exit
from the cranium in a similar way. The position of the brain differed
by the anterior lobes being more raised, in consequence of the project-
ing plates of the orbits internally, and by the posterior lobes and cere-
bellum lying lower than the human, according to the form of the base
of the cranium. This organ was not dissected.
The muscles and blood-vessels could not be so minutely examined, in
consequence of the warmth of the season, as to enable me to give a
correct account of them. The muscles were in general very distinct,
having their fasciculi of fibres remarkably strong. The blood-vessels*
were small. (Boston Journal of Philosophy, vol. ii.)
2. Comparison of Indian arid European Skulls.?Dr. Patterson,
of Calcutta, from a comparison of numerous skulls of Indians with
those of Europeans, has deduced that the head of the former is to that
of the latter race, as two to three. Or otherwise, that the head of an
European fifteen years of age, is of the same size as the head of an
Indian thirty years of age. ( Revue Ency.)
PHYSIOLOGY.
3. Experiments on Poisoning.?M. Segalas communicated to the
Academy of Medicine the result of some experiments made by him,
tending to prove that poisons rather produce their effects through the
medium of the vessels, than of the nerves. The following is the result
of his researches:?
Medical and Physical Intelligence. 517
1st. Having cut the spinal marrow of an animal, so as to render it pa-
ralytic, and having placed some alcoholic extract of nux vomica in the
paralysed parts, he perceived that tetanus came on just as quickly and
powerfully as if the nervous system had been entire.
2d. Having, on the contrary, left the spinal marrow untouched, but
prevented the blood which returned from the part where the poison had
been lodged from being carried to the heart, he observed that the poi-
soning did not take place.
3d. Tetanus appeared to come on equally quickly when he injected
the poison into the bronchi?, although the eighth pair of nerves were
divided.
4th. The nux vomica placed in the thigh of an animal rendered pa-
ralytic by the division of the spinal marrow, produced tetanus not only
in the trunk and upper extremities, but also in the paralysed parts.
5th. The same result takes place in whatever part the poison has
been placed; only the contraction of the paralysed muscles is slower,
and seems only to occur in proportion as the blood conveys the poison-
ous matter to the nerves which animate them.
6th. Having injected the poison into the crural artery of a paraplegic
animal, its effects were manifested in the like manner: the convulsions
commenced in the thighs, and only became general after the lapse of
time judged to be necessary for the conveyance of the poison to the
spinal marrow.
M. Segalas concludes from his experiments, that the voluntary mus-
cles can contract themselves, in certain cases, independently of the
action of the spino-cerebral system.
In these experiments, M. Segalas has often designedly made the divi-
sion of the spinal marrow at different points, but most commonly on a
level with the last vertebrae of the neck, or the first of the lumbar ver-
tebrae; and this has produced no modification of the phenomena.
(Archives Generates.)
4. On the Utility of Blood-letting.?M. Gibert, of Paris, in a
Memoir upon the above subject, observes, that local blood-letting is
preferable to general, in inflammations of membranes. As a proof, he
relates the case of a young man attacked with pleurisy, who had been
bled copiously from the arm five times in the first three days of the dis-
ease, without effect; but who was promptly relieved by the application
of a number of leeches to the pained part.
In the second part of his Memoir, M. Gibert says, that in many dis-
eases local bleeding is not only useless, but hurtful; and he recites, in
confirmation of this opinion, two cases where the disease was aggravated
by its employment. One was of an eating ulcer of the nose; the other,
a schirrous tumor of the breast.
M. Gibert relates some instances where bleeding has been found an
heroic remedy ; aud, amongst others, that of a patient who appeared to
be at the point of death, but who was restored by the loss of fourteen
palettes of blood, which was again practised in the evening and the
following day. (Archives Generates.)
518 Medical and Physical Intelligence.
PATHOLOGY.
5. Abscess of the (Esophagus.?A man, fifty-five years of age,
powerfully athletic, experienced a violent fit of rage, during which he
made great muscular exertion. Three days afterwards, he complained
of inability to swallow even liquids, and attributed it to a violent pain
on the left side of the larynx. No lesion, however, appeared on the
outside of the throat.?Twenty leeches were applied.
Next day, deglutition was quite impossible ; liquids returned through
the nose. A slight redness was perceived at the bottom of the throat, but
the swelling of the parts was not sufficient to hinder deglutition.?Bled
largely from the feet.
On the third day, great thirst, extreme agitation, and pain in the
neck excessive.?Again bled from the feet to sixteen ounces, and a
warm bath used. This brought on a convulsion, which lasted half au
hour.
Fourth day, symptoms still increasing.?Twenty leeches to the
neck, and ice to the head.
Fifth.?Another convulsion, still more severe than the last, with
every symptom of cerebral congestion. Head burning hot; face
red and swollen; conjunctiva injected; respiration feeble; pulse
depressed; suspension of all his faculties; deglutition still imprac-
ticable.?These symptoms yielded to the application of forty leeches to
the neck, sinapisms to the feet, a blister to the nape of the neck, and
ice on the head.
Sixth.?A gum elastic sound was tried to be introduced into the sto-
mach, ineffectually: it only reached the inferior part of the pharynx,
and there caused such violent pain, that it was obliged to be withdrawn.
Gargles were used to queuch thirst, and lavements of broth.
' This state was extended to the seventeenth day: then the patient
suddenly spit up by the mouth, and without effort, four spoonsful of
thick pus, bloody, and of an extreme fetor.
Eighteenth day, he had hiccup, followed by a peculiar sensation in
the stomach aud oesophagus, and by a very acute colicy movement
through the intestinal canal; and he soon passed by stool some
matter, mixed with bile, and exhaling an insupportable smell. There
was no return of this last evacuation, but that from the mouth conti-
nued fifteen days. Deglutition, at first difficult, was re-established by
degrees, and at the end of a month the cure was complete.
This appears to have been a case of abscess in the side of the oeso-
phagus, probably occasioned by the rupture of some of the muscular
fibres of that canal during the violent muscular efforts made when in a
furious passion. The cerebral symptoms were occasioned by the
pressure of the inflamed pharynx on the internal jugular veins. (Revue
Medicate.)
Melanoid Tumor of the Face.?In the Clinical Reports of the
Hospital de la Piti6, M. Ricord relates two cases; one of " cancer
melanique" of the face, which wds operated on, and followed by en-
largements of the submaxillary glands, with lancinating pains, &c.j and
6
Medical and Physical Intelligence. 51<)
which was cured by repeated applications of leeches, and strict attention
to diet.
In the second case, a cancer of the right breast had been removed,
and fifteen months after was followed by schirrous enlargement of the
glands and cellular tissue in the armpit. The means used were general
and local bleedings, belladonna, and, to relieve an increased action of
the heart, twelve grains of digitalis in a lavement, which had the de-
sired effect. In tJiis case, four leeches were applied to the upper and
inner part of the thigh, generally with the effect of bringing on men.
struation; and at one time, by being repeated during eight days, they
took place of the menses altogether. (lb.)
7. Observations on Salivary Calculi.?Calculous concretious are
occasionally produced from the saliva, as well as from the other animal
fluids. Fourcroy was of opinion that salivary calculi consist of
phosphate of lime and an animal substance, and that they are formed
when the saliva is supersaturated with the earthy salt. It is difficult to
comprehend in what manner, and under what circumstances, such a
supersaturation of so limpid a fluid should arise. Salivary calculi may
exist in any part of the cavities destined for the separation and passage
of the saliva. The histories of the cases on record are deficient in a
symptomalogical and diagnostic point of view, and, in respect to the
treatment of this interesting form of disease, but little has been said.
Elizabeth Hinzen, a robust and healthy peasant girl, sought the ad-
vice of Professor Walt her on account of a fistulous ulcer on the
upper part of the left side of the neck, at the bottom of which could
be detected, with a probe, a hard and somewhat moveable substance.
She had not previously suffered from any severe disease; she had men-
struated regularly, and could assign no probable cause for her present
complaint. It had arisen in the following mannerNearly three years
before, under the tongue towards the left side, a painful swelling had
made its appearance, which was very distressing during the act of mas-
tication. In the course of three days it had become of the size of a
hazel-nut; and, by frequent fomentations with warm milk, it broke into
the cavity of the mouth, and discharged a tolerable quantity of pus.
This swelling did not discharge its contents through the natural opening
of the Whartonian duct, but close to it. The discoloration of the
ulcerated spot could yet be perceived. Three months afterwards, the
swelling again occurred, with precisely the same appearances, and rup-
tured on an adjoining part. The suppuration lasted a long time, but
at length the ulcerated opening closed. The mark left was very per-
ceptible at a small distance from that of the first ulceration. Between
the two spots was the opening of the salivary canal, in which could be
easily introduced the point of a tine probe, to about the depth of two
lines. Some time after the healing of the second internal ulcer, arose,
on the left and upper side of the neck, near the angle of the under jaw,
a very painful swelling, of considerable size, which, by the employment
of warm emollient poultices, broke, and discharged a great quantity of
matter. This ulcerated swelling had since remained hstulous, and into
its cavity the two above-mentioned ulcerated openings led. Without
no. 328. 3 x
520 Medical and Physical Intelligence.
any success, the patient had employed various ointments, plasters, and
injections, in order to heal the fistula. When a probe was introduced
into the fistulous opening, at about the depth of an inch, it struck
against a hard resisting body, which appeared to have a rough surface,
and some degree of motion; although but an imperfect opinion could
be formed of its true nature. Without doubt, however, its presence at
the bottom of the fistulous passage was either the cause of the disease,'
or at least an impediment to the relief of the patient. It was also highly
probable that the external fistulous opening, and the two former ones
in the mouth, were dependent upon a common cause.
As the patient was very pressing lor the healing or the external open-
ing, which clearly depended upon the removal of the foreign body,
Walther proceeded, in the first place, to enlarge the fistulous passage by
means of a bistoury. The external maxillary artery was necessarily
divided. For a moment the hemorrhage was considerable: it was,
however, easily repressed by the pressure of the finger. It was not
possible to apply ligatures to the divided arteries, on account of their
depth. In the extirpation of the submaxillary gland, this difficulty is
not experienced. In this case, Walther has frequently tied these arte-
ries. In consequence of the enlargement of the gland, the artery is
raised, and lies in a more superficial situation. After thq widening of
the canal, the foreign body was with difficulty grasped and removed by
a pair of forceps. It proved to be a salivary calculus, of a long form,
about half an inch in size, and with a rough surface.
An uniting bandage was applied, and the patient put to bed. It was
necessary, however, to remove the bandage at the expiration of half an
hour, on account of the renewal of the hemorrhage. A piece of sponge
was now introduced into the wound, and remained till the beginning of
the fourth day, when it was removed. No more bleeding took place,
and in a short time the wound healed, after a moderate discharge of
laudable pus. The patient has since remained entirely well. (Gr'afe
und Walther, Journal jur Chirurgie.)
8. Spontaneous Discharge of Salivary Calculi from the Whartonian
Duct.?Graf L. St. G. a man sixty-four years of age, who had suffered
frequently from catarrhal, rheumatic, and hepatic affections, and who,
two years before, had been treated by Professor Walt her for a gan-
grenous and aphthous ulceration of the palate, had for sixteen years
been affected with a hard swelling of the left submaxillary gland. This
swelling arose without any obvious cause. It was considered as a
scrofulous enlargement, although the affected gland was not a lymphatic,
and the conglobate glands were free from enlargement. Throughout
his early years, also, the patient had been entirely exempt from any
strumous disease.
For the purpose of dispersing the enlargement, many remedies
were in vain employed. The enlarged gland felt as hard as a
true schirrus, and would certainly have been considered as such, and its
extirpation undertaken, if a particular circumstance had not occurred,
which is never observed in true schirrous swellings,?namely, a great
change in the size of the enlargement, which sometimes, without any
Medical and Physical Intelligence. 521
obvious cause, very much diminished, and indeed almost totally disap-
peared, and tlieu always regained its former volume. It became gra-
dually of the size of a hen's egg. Upon pressure, it was very painful,
and hindered the free motion of the lower jaw, and rendered the act of
swallowing difficult.?Leeches freely applied, and various cataplasms,
and other means, afforded but little benefit.
One morning the swelling was much diminished, and the patient con-
sequently greatly relieved. The pain and difficulty in swallowing were
much abated. Upon looking into the mouth, a foreign body was dis-
covered under the tongue, which had not been detected by the patient.
It was removed, and ascertained to be a salivary calculus, which had
spontaneously passed through the Whartonian duct, after having been
formed in the submaxillary gland. Upon drawing the recollection of
the patient to the previous state of the parts, he remembered he had
observed a small pimple under the tongue, which had been painful, and
broke within two or three days, discharging a clear fluid. This appear-
ance might probably have beerv caused by an obstruction of the opening
of the Whartonian duct, in consequence of the presence of a small
calculus, the removal of which would have been followed by an in-
creased discharge of the salivary fluid. The patient had never suffered
from Ranula. The opening of the Whartonian duct was not found to
be enlarged or torn, even immediately after the escape of this calculus,
which was rather large. The swelling now diminished in size; but, as
it still continued hard, it was apprehended that other calculous bodies
might be contained in the ramifications of the duct, in the substance of
the gland.
Some months after, another calculus escaped, lhe passage of it
through the canal, and its arrival in the mouth, were clearly felt by the
patient. The submaxillary gland still remained bard and enlarged.
The great object was now to effect a radical cure, and to prevent the
formation of more calculi; and, considering the length of time the pa-
tient had been afflicted, the complete success that resulted from the
internal use of the carbonated potass was hardly to be expected.
This medicine has been employed by Walther for many years, with
great advantage, to prevent the formation of calculi in the kidneys.
It had also been previously employed, with success, to hinder the
formation of lachrymal calculi in the space between the eyeball and
eyelids. The origin, not only of urinary calculi, but of all animal con-
cretions, is dependent upon a peculiar acid matter, which is formed in
the fluids of the human body in certain proportions, and which, uniting
with the first deposition, forms the origin of the concretion. The Graf
L. took, for more than half a year, daily about twelve grains of carbonate
of potass, dissolved in cinnamon water ; and subsequently no calculus
passed. The enlargement of the gland gradually diminished, and at
length totally disappeared.
Walther states, that he considered the continuance of the medicine
necessary for some years, in order to prevent the formation of calculi in
the Whartonian duct, as the effect of the alkalies in restraining the dis-
position to calculous depositions is not permanent. (Ibid.)
Many observations follow the detail of the above cases, which
522 Medical and Physical Intelligence.
cannot be considered without interest, but which appear to show that
the author is not acquainted with the labours of Prout, Marcet,
&c. &c. who have so fully explained the particular cases in which the
alkalies are likely to be of benefit, and the manner in which they
should be employed.
PRACTICAL MEDICINE.
9- Bicarbonate of Soda as a Lithontriptic.?M. Robiquet read
a notice on the employment of this salt in urinary calculi. Having
learnt of M. Daret, that the use of the waters of Vichy renders that
urine alkaline which was previously acid, he conjectured that this effect
was owing to the bicarbonate of soda which these waters contain, and
therefore conceived the idea of administering it internally. Last July,
he tried it in the case of a man, seventy-four years of age, who had
suffered from the February preceding, and in whom a stone had been
detached by sounding, small and slender, and capable of being extracted
by M. Civiale's process. He was ordered to take, during the day,
two litres of a solution of the above salt, in the proportion of five
grains to the litre, together with hip-baths, lavements, &c. Fifteen
days of this treatment produced great relief. At the end of a month,
the patient appeared quite cured ; nevertheless, the same treatment w&s
continued, and, last November, the man passed by the urethra a small
calculus of uric acid, which appeared to be the nucleus of one that had
been much larger, the external surface of which had been worn away.
The patient has not suffered since, but he has not been sounded.
(Archives Generates.)
10. Tincture of Dhatura.?Dr. Adam submitted to the Medical
Society of Calcutta a tincture of dhatura, which he had lately made;
conceiving that it would be found, in that country especially, a useful
form of exhibiting that remedy. It is a saturated tincture, prepared
.from the capsules, seeds, and leaves, mixed in equal parts, and with the
proportion of proof spirit used by the London College in the prepara-
tion of tincture of digitalis. The variety of the plant used is the
Dhatura fastuosa (kala dhatura), cultivated under his own inspection
at Allipore. This is reckoned more powerful than any of the other
species, and it was therefore selected on that account. The tincture is
of a dark green colour, and resembles in that respect, as well as in fla-
vour, the saturated tincture of digitalis. He has only yet employed it
in two cases,?one of asthma, in a native; the other of organic disease,
most probably aneurism of the aorta, in a European. It was given in
the dose of thirty and forty minims, repeated aftet two hours, to four
doses, when relief was obtained. (Transactions of the Med. and Phys.
Society of Calcutta.) /
STATISTICAL MEDICINE.
1!. Plague and Yellow Fever.?M. Renauldin, in the name of
a commission composed of twelve members, submitted to the Academy
the plan of an answer to be made to the Minister, on the subject of the
Medical and Physical Intelligence. 523
experiments that MM. Costa Lassis and Lassbre have proposed to
make in the lazaretto of Marseilles, to determine the non-contagion of
plague and yellow fever. After enumerating the dangers that might re-
sult to France from the introduction of the materials necessary to these
experiments, he concludes by proposing to government to accept the
offers of the experimenters, but only in the event of yellow fever or
plague being accidentally introduced into the lazaretto; and then to be
careful that the experiments are in a separate quarter of the lazaretto.
He compares the dangers that the experimenters wish to brave to those
which are encountered in the pursuit of geographical discoveries, and
which government not only permits, but encourages. (Revue Med.)
12. Diminution of Goitre in the Pyrenees.?When presenting a
Memoir on Goitres, by M. Roultn of Bogota, to the Academy, M.
Magendie, who has lately traversed the Pyrenees, stated that this
disease is much less frequent than heretofore. This amelioration, ac-
cording to him, is caused by the increased riches of the inhabitants,?
the extended culture of grain,?and by the better construction of the
houses. M. Moegey remarked on this subject, that M. Fabrq^i
thought he had observed that the granitic valleys only of the Pyreifees
were exempt from the goitre. (Ann. de Chimie.)
SURGERY.
13. Operation for Phymosis.?M. J. Cloquet related to the
Academy of Medicine a mode, which he regards as new, of performing
the above operation, and which has the advantage of leaving no de-
formity. This method consists in introducing a grooved director within
the prepuce on a level with the fraenum and parallel to it, and cutting
the prepuce at its lower part. If the fraenum is very short, it should
be divided with the scissars. The longitudinal wound thus made be-
comes transverse as soon as the prepuce is drawn back behind the
glans; so that the prepuce acquires in breadth what it had lost in
length. Several patients, on whom M. Cloquet had operated in this
manner, were so perfectly cured that it was difficult to see the cicatrices,
the prepuce appearing to have its natural conformation. (Archives
Generates.)
MIDWIFERY.
14. Impregnation in the Fallopian Tube.?By order of the
civil authorities, Professor G. 8. was called upon to examine the
body of a healthy robust unmarried female, eighteen years of age, who
had died suddenly. No external lesion or alteration was visible; the
members were all flexible; a large ecchymosis occupied the umbilical
region, the left hypochondrium, and corresponding thigh. An incision
was made in the parietes of the abdomen, and the first appearance that
presented itself was a considerable quantity of coagulated blood, which
filled the cavity, especially the left hypochondrium, aud the correspond-
ing part of the pelvis. Many clots of blood were also found in exa.
524 Medical and Physical Intelligence.
mining the condition of the viscera, and among these a ball, of about
twice the size of a goose's egg, was extracted, formed of transparent
membranes, through which was distinctly perceived a foetus of the male
sex, floating in its proper fluid, apparently of about four months'
growth. The internal organs of generation being next examined care-
fully, and cleansed from the coagulated blood, a rupture of the left
fallopian tube was discovered, where the above-mentioned foetus had
been lodged. This tube was rent in its central point, to which the
placenta adhered. To the division of the parietes of the distended tube
was also added that of the little umbilical cord, from whence arose the
hemorrhage, the falling of the ovum into the cavity of the abdomen,
and the death of the mother.
The uterus being separated, and accurately examined, it appeared
rather more voluminous than in the virgin state, as well as redder,
softer in substance, and having its internal surface covered with a floc-
culent membranous stratum, spongy, and of a yellowish white colour.
The right fallopian tube was rather more distended and more vascular
than natural. Both the ovaries were enlarged, and the right contain-
ing a corpus hiteum, the left spermatic artery and vein were of
a much larger calibre than ordinary. The left fallopian tube had lost
ils natural shape, and appeared like a thin membranous sac, interwoven
with many blood-vessels much increased in size : its free extremity em-
braced the left ovarium. Below the distended part, towards the uterus,
the tube was entirely obliterated.
On examining the ovum and foetus, it was found that the placenta
was not of a size corresponding to that of a foetus of four months. The
foetus and membranes were natural, as was also the position and attach-
ment of the umbilical cord. (Annali TJniversali.)
15. Extract of a Letter from T. E. Baker, Esq. of Buxar, de-
scribing a singularly small Child.?The following account of a singu-
larly small child, you will probably think sufficiently interesting to
communicate to the Society. Some of the members will probably be
able to inform me whether there is any well authenticated history of an
equally small child, having ever lived more than a few days.
The child is the daughter of a Mrs. Green, the wife of the riding-
master of the 5th Native Cavalry, and is now, with the mother, living
at this station with Mr. Edwards, an overseer to the Honourable Com-
pany's stud dep6t. It has been seen by Mr. Surgeon Gibb, the super-
intendent of the stud; by Mr. Thompson, the civil surgeon at Arrah;
by Captaiu J. Mackenzie, and other residents at the station.
The mother was coming by water from Agra, and was confined near
Bandah, when she thought herself about six months and a half gone
with child; and attributed her premature confinement to having over-
exerted herself in removing some boxes, &c.
On this day (May 24th), the child is one month and twenty days old;
it weighs exactly one pound and thirteen ounces, and is fourteen inches
in length. The following are the dimensions of the principal parts of
the body:
Medical and Physical Intelligence. 525
Circumference of the head (longest diameter) . 10 inches.
Ditto ditto (shortest diameter) . 9 inches 1-lOth.
Ditto of the chest . . . . 9 inches.
Ditto of the body . . . . 8 inches.
Ditto of the thigh, midway between the knee
and the hip-joint 2 inches 6-10ths.
Ditto of the fore-arm, midway between the^
wrists and elbow . . . . . .1 inch 7-10ths.
I much regret the weight and dimensions of this child were not taken
when it was first born ; for the mother informs me it has grown consi-
derably since that lime. At first it would not take to the breast, but
now it sucks very well.
The bones of the head are rather loose, and the anterior aud posterior
fontanels are large in proportion to the size of the head.
The mother of the child is a healthy womanr, aud has four other very
fine children.
It is, of course, very uncertain whether this child will live; but, should
it be alive when three months old, I shall again take the dimensions of
it. (Trans, of the Med. and Phys. Society of Calcutta.)
CHEMISTRY.
16. Account of Prof. Berzelius's Mtthod of detecting Arsenic in
the Bodies of Persons poisoned.?Prof. Berzelius has lately given some
instructions for the discovery of arsenic in persons that have been poi-
soned with it. He considers the reduction of arsenic to the metallic
state as ,the only incontestible proof of the presence of this poison.
Arsenic may occur in two ways,? viz. when it is found in substance (iu
the state of arsenious acid') in the dead body, and when it is not found
in this state; though the intestines of the dead body may contain it in
the state of a solution.
In the first of these cases, it is easy to determine the presence of
arsenic. In order to do this, take a piece, about three inches long, of an
ordinary barometer tube, and having drawn out one end of it into a
much narrower tube, close the end. Let some of the arsenic found in
the body be now put in at the open or larger end, so that it may fall
down to the bottom. Any quantity of this arsenic, of sufficient volume
to be taken from the body, will suffice for this purpose. The arsenic
being at the bottom of the small part of the tube, a little charcoal is let
fall upon it, after it has been freed from all moisture by bringing it to a
red heat with the blowpipe. The charcoal is then heated in the tube at
the flame of a spirit-lamp, the point where the arsenic lies being held
out of the flame. Wheu the charcoal is very red, the point containing
the arsenic is drawn into the flame. The arsenic is then instantly vola-
tilised, and, passing into vapour by the red charcoal, it is reduced, and
reappears on the other side of the flame in a metallic state. The flame
is then brought slowly towards the metallic sublimate, which is thus
concentrated into a smaller space in the small tube; and then presents
a small metallic ring, shining like polished steel.* We have now only
* Had the experiment been made in the wide part of the tube, the result would
scarcely have been visible with a small quantity of arsenic.
526 Medical and Physical Intelligence.
to verify, by its smell, that the metallic sublimate is arsenic. For this
purpose, cut the small tube with a file a little above the sublimate, and,
having heated the place where it lies, put the nose above it at a small
distance, and the particular odour of the metal will be immediately
perceived.
In the case where the solid arsenic cannot be found, we must collect
as much as possible of the contents of the stomach and the intestines, or
even cut the stomach in pieces, and mix it with its contents. The
whole is then to be digested with a solution of hydrate of potash.
Hydrochloric acid is then added in excess. The whole is filtered, and,
if the liquor is too much diluted, it is concentrated by evaporation. A
current of sulphuretted hydrogen is then passed through it, which pre-
cipitates the arsenic in the form of the yellow sulphuret. If the quan-
tity of arsenic is very small, the liquid will become yellow without
giving a precipitate. It must then be evaporated; and, in proportion
as the hydrochloric acid becomes more concentrated, the sulphuret of
arsenic will begin to be deposited. It is then filtered. If the sulphuret
remaining on the filter is in too small a quantity to be taken from the
paper, add some drops of caustic ammonia, which will dissolve it.
Then put the liquid which passes the filter into a watch-glass, and eva-
porate it. The ammonia will be volatilised, and will leave as a residue
the sulphuret of arsenic. If it shall still be difficult to collect the sul-
phuret, we must put into the watch-glass a little pulverised nitrate of
potash, and, with the finger, mix the sulphuret with the nitrate of pot-
ash, which detaches it from the glass. At the bottom of a small phial,
or a piece of glass tube, shut at one end, melt a little nitrate of potash
at the flame of a spirit-lamp, and introduce into it, when melted, a
little of the mixture which contains the sulphuret of arsenic. It is
oxidised with effervescence, but without fire or detonation, and without
loss of arsenic. The melted salt is then to be dissolved in water, and
lime added in excess, and the liquid boiled. The arseniate of lime will
then be deposited, and may be collected. When dried, it is mixed
with charcoal, and then brought to a red heat by the blowpipe; and a
small quantity of this mixture is allowed to fall to the small end of the
above-mentioned tube. It is now gradually heated to expel all humidity
which tends to throw it into the wide part of the lube; and, when it is
very dry, heat, at the flame of the blowpipe, the part of the tube which
contains the mixture. The arsenic will be disengaged, and be sublimed
at a distance from the heated part. An addition of vitrified boracic
acid greatly promotes the decomposition which then takes place at a
less elevated temperature; but the acid frequently contains water, and
produces a bubbling of the melted matter, which raises it in the tube,
and causes the vapours to issue by perforating the softened part of the
glass.
M. Berzelius maintains, that the sixth part of a grain of sulphuret
of arsenic is sufficient to make three different trials; but he add3, that,
when we have discovered only very small traces of arsenic, we must
take care not to introduce any by means of re-agents, among which,
both the sulphuric and the hydrochloric acid may contain it. The first
almost always contains some arsenic when it is manufactured from vol-
Medical and Physical Intelligence. 517
canic sulpur; and the second, in consequence of sulphuric acid being
used in the preparation of hydrochloric acid, yields the arsenic which it
contains in separating it from soda. We must, therefore, be certain of
the purity of these reagents.
When death has been caused by the arsenic, and not by the arsenious
acid, the process must be modified, because the sulphuretted hydrogen
gas decomposes the arsenic acid too slowly. In this case, we must add
hydrosulphuret of ammonia, which reduces the arsenic acid to the state
ofsulphuret, which is afterwards precipitated by the hydrochloric acid.
(Edinburgh Phil. Journal.)
MEDICAL JURISPRUDENCE.
17. Marks of Vitality in New-born Infants.?It was in pursuing
these researches that Dr. Bernt hit upon an observation, which, if
correct, will establish the distinction of an inflated from an uninflated
lung, upon the most solid foundation. He seems himself to consider it
as an almost infallible test.
The circulation of a child undergoes an extensive revolution the mo-
ment it breathes, and the placental subsidiary action becomes suspended.
The blood arriving at the liver by the umbilical vein, is withdrawn from
that organ, which ought to be rendered so much lighter by its abstrac.
tion; and the lungs, by the blood of the ductus arteriosus flowing
through them, must have become proportionally heavier. The mere
examination of these circumstances in a new-born child, will go far to
determine whether it has breathed ; but the test which he chiefly rests
upon, is the appearance and situation of the opening of the foramen
ovale. This hole, in a child which has never breathed, is exactly in the
bottom of the fossa oralis; but, as soon as the child has breathed, the
aperture becomes turned towards the right; in several weeks, it has
ascended very high ; and, in adult age, is found to have arrived at the
summit of the oval. In other words, from the moment respiration
commences, the aperture of the foramen ovale begins to travel from the
lower extremity of the oval hole towards the upper, proceeding from
left to right: and the degree of advance it has made, becomes an index
of the existence and duration of the respiratory process.
This semi-revolution of the aperture around the centre of the axis of
the foramen ovale, after birth, so important in an anatomical and phy-
siological, as well as docimastic, point of view, is in some degree hinted
at by our countryman, Dr. Humphry Ridley, (Ohserv. de Cord.
Embryon. Lugd. J750, at p. 180?4;) but afterwards more fully de-
veloped by Dioboldt, in 1771.
The reason of the phenomenon, Dr. Bernt conjectures to be the con-
traction of the muscular fibres of the isthmus Vieussenii; but we are
ourselves inclined to attribute it rather to the diminished resistance of-
fered, after birth, to the stream of blood projected from the right ven-
tricle, and the consequently increased resistance to that which is
simultaneously sent from the left. It is known that accumulation in the
right ventricle generates also an accumulation of blood in the right au-
no. 328. ' 3 y
528 Medical and Physical Intelligence.
ricle, and even in the venae cavae which open into it. As long as this
state continues, the lower cava, aided as it is by the placental contribu-
tion, will be predominant; and, ascending from below, will keep the
valve of the oval hole from adhering to the edges, from above down-
wards; or, in other words, will preserve the aperture at the bottom of
the fossa. But, as soon as the aortal resistance is withdrawn from the
right side of the heart, by respiration, and the placental supply from the <
lower cava, by obliteration of the cord, the equilibrium becomes almost
perfect between the ascending and descending cava, in the infant; and
the centre of their joint impulse must be brought up near, or beyond,
the central diameter of the oval hole. The valve, however, can only be
displaced gradually in this direction; and, accordingly, it is not before
the adult age that the aperture arrives at the top of the valve. This
ascent is much assisted by the preponderance of the left ventricle, which,
reacting against the whole resistance of the circulation, lessens the angle
of position of the axis of the right heart to the axes of the hollow veins,
and thereby the line of pressure upon the oval hole, in the direction up-
wards. At all events, whatever is the theory, the fact is most valuable;
and we consider both the docimastic methods above adduced, as afford-
ing simple, easily practicable, and therefore most valuable, additions to
the jurisprudential part of our profession; and to that part of it which
is never exercised without great hazard of the reputation, and certain
laceration of the feelings, even to the most conscientious practitioners.
(Edinburgh Journal of Medical Science, No, 2.)
NATURAL HISTORY.
18. Fecula of Plants.?M. Raspail read a Memoir on the Deve-
lopment of the Fecula of Plants. His conclusions are, that it exists
always free in the cells of plants; that, observed by the microscope, it
appears in the form of round grains, hard and translucid, of different
diameters; spherical in the orchis and in the cerealia; irregular in the
potato, and much larger than in other plants. The diameter of the
grains of fecula increase with the age of the plant. The blue colour
which it assumes with iodine operates no change in its form : it may be
deprived of its colour by an alkali, and coloured again by the iodine,
a great many times, without undergoing any change. These grains are
composed of an exterior integument, of an internal substance, analogous
to gum, and solid at the common temperature of the air: when heated,
'>**they increase in size, and the'interior substance makes its way through
the integument, either by tearing it or by passing through its tissue.
When boiled in>a quantity of water, the gummy matter is dissolved,
whilst the integuments are precipitated on cooling. They are white,
and unchangeable even ly the concentrated acids. (Archives Gen.)
MISCELLANEOUS.
19- Breeding of Leeches.?M. Chatelain, chief apothecary to
the navy at Toulouse, uses this method:?He places the leeches into
Catalogue of Medical Books. 529
earthen vessels, at the bottom of which a stratum of clay, made into a
paste, is put, which should be separated from the sides of the vessels a
little all round, that the leeches, in penetrating to the bottom of the
vessel, may be able to rise again. M. C. also recommends them to be
kept in places not too cold: they require the atmospheric heat of spring
to deposit their eggs.
M. Guibort says, that, as the clay soils the leeches, it would be
better to put pure sand at the bottom of the vessels ; placing a tube so
that, being plunged to the bottom, fresh water might by degrees be
added : in this way the old water would escape by the top of the vessel,
and the leeches might always have fresh water. M. G. thinks that the
custom of changing at once the whole mass of water in which leeches
have been, kills a great many of them, from the sudden change of
temperature.
On this subject, M. Peli.etier cites an interesting factSome
carp, which were living in the corrupted water of a tank, were seized
with an eruptive disease, (reddish pustules,) which caused them to die.
He threw some animal carbon into the water, which became good, and
the fish recovered their health. Some authors have looked upon this
disease of the fish to be a species of small.pox. ( Revue Medicate.)
530
METEOROLOGICAL JOURNAL,
From April 20, to May 19, 1826, inclusive.
By Messrs. William Harris and Co. Mathematical Instrument Makers,
50, Holboru, London.
Apr.
20
21
22
523
24
25
26
27
28
29
30
May
1
2
3
4
5
6
7
8
9
10
11
12
13
14
15
16
17
18
19
Raiu
gauge
.10
.17
29.74
29.54
29.50
29.65
29.76
29.88
29.56
29.35
29.75
29.88
30.01
30.15
30.00
29.91
29.94
29.90
29.90
29.93
29.93
29.94
29.95
29.96
30.10
30.15
29.98
29.99
29.99
29.98
30.00
2y.78
29.54
29.54
29.62
29.72
29.82
29.73
29.53
29.62
29.84
29.98
30.12
30.08
29.92
29.97
29.94
29.98
29.93
29.93
29.94
29.93
29.93
30.05
30.16
30.04
29.98
30.00
29.95
30.00
29.89
29.62
UE
LUC's
HYG.
9 A.M.
10p.m.
ENE
ssw
SSE
WNW
NNW
WNW
NNW
SSE
NW
N
N
N
N
NE
NNE
NNE
NNW
NEva.
ENE
NE
NE
NNE
ENE
NNE
ESE
NW
W
sw
sw
ENE
NNW
WNW
NNW
W
ENE
NNW
WNW
N
NNE
N
SE
NNE
N
N to E
NNE
NNE
NE
SE
ESE
E
NE
E
E
SE
wsw
WNW
sw
ESE
ATMOSPHERIC
VARIATIONS.
9 A.M.
Fine
Cloud.
Fine
Fair
Rain
Faii-
Cloud.
Faii-
Cloud.
Fine
S. Rain
Fiue
2 P.M.
Fine
Fair
Fine
Raiu
Fair
Rain
Fine
Fair
Fine
Rain
Fair
Rain
Fine
Rain
Fine
The quantity of rain fallen in the month of April,
was 76.100ths of an inch.

				

